# Prognostic factors of metastatic myxoid liposarcoma

**DOI:** 10.1186/s12885-020-07384-1

**Published:** 2020-09-14

**Authors:** Yusuke Shinoda, Eisuke Kobayashi, Hiroshi Kobayashi, Tomoaki Mori, Naofumi Asano, Robert Nakayama, Hideo Morioka, Shintaro Iwata, Tsukasa Yonemoto, Takeshi Ishii, Tohru Hiruma, Akira Kawai, Hirotaka Kawano

**Affiliations:** 1 Department of Rehabilitation Medicine, The University of Tokyo Hospital, Tokyo, Japan; 2 Department of Orthopaedic Surgery, Faculty of Medicine, The University of Tokyo, 7-3-1 Hongo, Bunkyo-ku, Tokyo, 113-0033 Japan; 3 Division of Musculoskeletal Oncology, National Cancer Center Hospital, Tokyo, Japan; 4 Department of Orthopaedic Surgery, Keio University School of Medicine, Tokyo, Japan; 5 Division of Orthopaedic Surgery, Chiba Cancer Center, Chiba, Japan; 6 Division of Musculoskeletal Tumor Surgery, Kanagawa Cancer Center, Yokohama, Kanagawa Japan; 7 Department of Orthopaedic Surgery, Teikyo University School of Medicine, Tokyo, Japan

**Keywords:** Myxoid liposarcoma, Metastasis, Prognostic factor

## Abstract

**Background:**

Myxoid liposarcoma (MLS) has the tendency to metastasize extrapulmonary. Although prognostic factors at the initial diagnosis of MLS have been reported, those at diagnosis of metastasis remain unclear. The purpose of this study was to investigate the prognostic factors for disease-specific survival at the initial diagnosis of metastasis.

**Methods:**

This retrospective observational study was conducted at three cancer centers and two university hospitals in Japan. Of 274 MLS patients pathologically diagnosed between 2001 and 2015, 48 metastatic patients were examined.

**Results:**

Lung metastases were detected in nine patients (18.8%) and extrapulmonary metastases in 45 (93.8%). Interval from primary diagnosis to the first metastasis was significantly shorter in patients with lung metastases than without (*p* = 0.007). Median disease-specific survival after diagnosis of metastases was 52.5 months in all patients. In multivariable analysis, liver metastasis (hazard ratio (HR), 2.71 [95% confidence interval (CI), 1.00–7.09]) and no evidence of disease (NED) achieved by radical treatment (resection with or without radiation therapy, or radiation therapy ≥60 Gy) or semi-radical (radiation therapy ≥40 Gy) treatment were significantly related to survival (HR, 0.36; 95%CI [0.13–0.95]). The number of metastases (odds ratio (OR), 0.44; 95%CI [0.25–0.78]) and abdominal/retroperitoneal metastases (OR, 0.09; 95%CI [0.008–0.95]) were the significant inhibitory factors of achieving NED.

**Conclusions:**

This is the first study to statistically demonstrate the importance of achieving NED with surgical resection or radiation therapy for longer survival in metastatic MLS patients. As number of metastases was a significant factor for achieving NED, early detection of metastases might be important.

## Background

Myxoid liposarcoma (MLS) is the second most common type of liposarcoma, representing approximately one third of all liposarcomas and 10% of all adult soft tissue sarcomas [1, 8]. MLS carries an intermediate risk with approximately one third of patients developing metastases and eventually dying of these tumors [5, 11, 14, 23].

Factors found to influence the prognosis at initial diagnosis of MLS include patient age, tumor size, tumor depth, the surgical margins achieved, and morphological factors such as grading, necrosis and mitotic rate, proliferation index (MiB-1, Ki-67 immunostain), and P53 overexpression [5, 9, 11, 14]. Of these, the amount of round cell component is reported to be the most important factor affecting the development of distant metastases or survival [2, 9, 12, 14, 16].

MLS has been reported to have a characteristic metastatic behavior; the incidence of extrapulmonary metastases, including the trunk and extremities, is as high as 87% of the metastatic MLS patients [10]. Although some studies have investigated the metastatic patterns of MLS, the prognostic factors related to metastasis characteristics at the initial diagnosis of metastasis remain unclear. Spillane et al. [24] have recommended aggressive treatment for metastatic disease involving further surgery; however, a treatment strategy for metastatic MLS has yet to be established.

In this retrospective, multi-center study, we enrolled MLS patients with metastasis, examined the characteristics of the metastases, and investigated the prognostic factors affecting overall survival at the initial diagnosis of metastasis. Then, we discuss a treatment strategy for metastatic MLS.

## Methods

### Study design and setting

This was a retrospective, observational study conducted at three cancer centers and two university hospitals in Japan (Higashi-nihon Orthopedic and Pediatric Sarcoma Group; HOPES); the study was performed in accordance with the Declaration of Helsinki and ethical guidelines for epidemiological research of the Japanese Ministry of Education, Culture, Sports, Science and Technology and the Ministry of Health, Labor, and Welfare.

### Participants

Between 2001 and 2015, a total of 274 patients with MLS were pathologically diagnosed and treated at these hospitals. All MLS patients were prospectively registered in a computerized database. There were 260 (94.9%) M0 patients and 14 (5.1%) M1 patients at initial diagnosis. We regarded patients as M1 if the metastasis had been detected within 1 month of initial diagnosis. Of the M0 patients, 38 patients developed metastasis during follow-up. Basically, whole body CT was performed at least once a year until 5 years after the initial diagnosis. But 2 patients were followed by plain chest radiography only, and 5 were followed at other hospitals.

We extracted the data from a total of 52 (19.0%) patients with metastasis; however, we excluded four patients whom we could not follow up after the diagnosis of metastasis.

### Data collection

We extracted the following clinic-pathological data collected at the time of initial metastasis diagnosis from the database: age; sex; time to metastasis (defined as interval from primary diagnosis to the first metastasis. It was defined as 0 month in M1 patients); number of metastases; location of each metastasis; diameter of the largest metastasis; and modality used for detecting metastasis. In addition, we examined the treatment details for the metastasis. We defined local radical treatment as R0 resection, R0 or R1 resection with adjuvant radiation therapy, or radiation therapy with ≥60 Gy, including carbon ion therapy and photon beam therapy, and semi-radical treatment as radiation therapy with ≥40 Gy. We next defined status following local treatment; no evidence of disease (NED) signified the status after completion of radical or semi-radical treatment for all of the remaining tumor. Remaining tumor included all metastases, primary tumors, and local recurrence, detectable in imaging examination. While alive with disease (AWD) signified any remaining lesion with or without palliative therapy. In addition, we examined the prognosis at final follow-up (NED, AWD, or dead of disease [DOD]). NED at the final follow up was defined as status with no active disease detectable by imaging inspection. Finally, we analyzed the prognostic factors for overall survival following the initial diagnosis of metastasis.

Regarding primary tumor, we also extracted the following data: age at diagnosis of primary tumor, location, size, if radical treatment or adjuvant chemotherapy were done, and whether there was a local recurrence. For NED patients, we also examined if the patients received adjuvant chemotherapy.

### Statistical analysis

The duration from primary diagnosis to metastasis and cumulative survival rates were calculated using the Kaplan-Meier method. The difference in time to metastasis by existence of lung metastases was analyzed using the log-rank test. Survival following diagnosis of metastasis was defined as the time to disease specific death. Prognostic factors were identified by Cox-proportional hazards regression analysis. A *p-*value < 0.05 was considered statistically significant. Variables revealed to be significant by univariate analysis were evaluated by log-rank test and multivariable analysis using Cox-proportional hazards model. Statistical analyses were performed with JMP 13 (SAS Institute Inc., Cary, NC, USA).

Factors for achieving NED were analyzed by logistic regression analysis. Factors revealed to be significant by univariate analysis were examined using multivariable analysis.

## Results

Median follow-up following primary diagnosis was 47.4 (4.7–187.2) months and that following initial metastasis was 25.8 (2.1–96.8) months. The baseline clinical characteristics at the initial diagnosis of metastasis are shown in Table 1. Thirty-four patients (70.8%) were male and the median age was 43 years. The number of patients with metastasis at primary diagnosis (M1 patients) was 14 (29.2%). The median duration from primary diagnosis to metastasis was 27.5 months for M0 patients, and 13.2 months for all patients including M1. Twenty-three patients (47.9%) had multiple metastases and 45 patients (93.8%) had extrapulmonary metastasis. Three patients (6.3%) had only lung metastasis, six patients (12.5%) had both pulmonary and extrapulmonary metastases, and 39 patients (81.3%) had only extrapulmonary metastases. The median size of the largest metastasis for each patient was 5 cm and eight patients (17.8%) had a metastasis > 10 cm. Thirty-five patients (72.9%) were diagnosed with metastasis by computed tomography (CT), 13 (27.1%) by magnetic resonance imaging (MRI), and one by positron emission tomography (PET)-CT.

**Table 1:** Baseline characteristics at the initial diagnosis of metastasis (*N* = 48)

			^a^, ^b^, ^c^, ¶
Variable	n	%	
*Sex*			
Male	34	70.8	
Female	14	29.2	
*Age* [median: 43 years (26–72)]			
< 45	26	54.2	
≥ 45 years	22	45.8	
*Time to metastasis*^a^ [median: 13.2 months (0–140.1)]			
**at primary diagnosis (M1)**	**14**	**29.2**	
**during follow up (M0) [median: 27.5 m]**	**34**	**70.8**	
< 24 months	16	33.3	
≥ 24	12	25.0	
≥ 60	6	12.5	
*Number of metastasis at the initial diagnosis of metastasis*			
single	25	52.1	
multiple	23	47.9	
*Location of metastasis*^b^ *(number of patients)*			
**lung**	**9**	**18.8**	
**extrapulmonary metastasis**	**45**	**93.8**	
bone	15	31.3	
abdomen or retroperitoneum	11	22.9	
liver	9	18.8	
other soft tissues^c^	23	47.9	
*Size of the largest metastasis* [median: 5 cm (1–20)]			
< 10 cm	37	82.2	
≥ 10	8	17.8	
*Diagnostic modality for the first metastasis*			
CT with contrast enhancement	22	45.8	
CT without contrast enhancement	13	27.1	
MRI	11	22.9	
PET-CT	1	2.1	
Others	1	2.1	

^a^ Interval from primary diagnosis to the first metastasis

It was defined as 0 month in M1 patients

^b^ Presence of metastasis in indicated organ with or without that in another location

^c^ including lymph node metastasis

Abbreviations: *CT* computed tomography; *MRI* magnetic resonance imaging; *PET-CT* positron emission tomography

Regarding the primary tumor, characteristics and treatments were shown in Supplementary Table. 1. In M0 patients, primary tumor was resected in all of the patients, and adjuvant chemotherapy was performed in 6 out of 34 patients (17.6%). In M1 patients, resection was performed in 7 patients (50.0%).

**Fig. 1:**
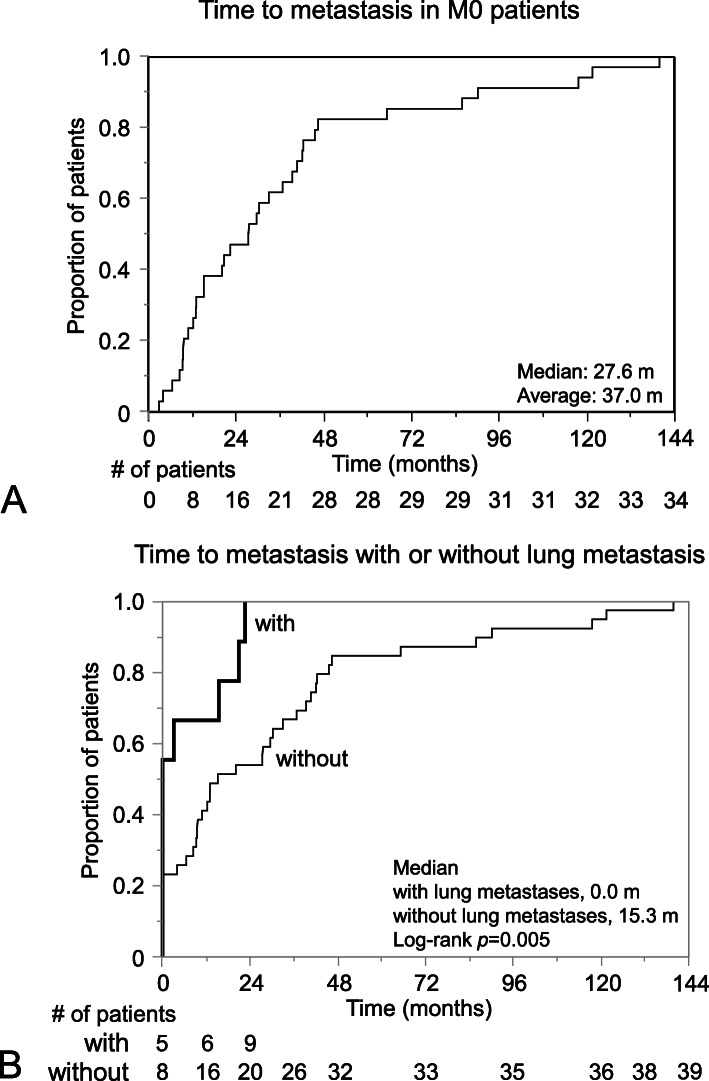
Time to metastasis from the primary diagnosis. **a** M0 patients (*n* = 34), **b** all patients with or without pulmonary metastases (*n* = 48). **a** Twenty-eight patients (82.4%) developed metastasis within 4 years and 31 patients (91.2%) within 8 years. Only three patients (8.8%) developed metastasis after 8 years of follow-up. **b** “with lung metastasis” means the presence of lung metastasis with or without metastasis in another location, and “without lung metastasis” means absence of lung metastasis at the initial diagnosis of metastasis. In patients “with lung metastases”, five out of 9 patients (55.6%) had metastasis at the initial diagnosis of primary MLS, and all patients were diagnosed to have metastasis within 2 years, which was significantly shorter than in patients “without lung metastases” (15.3 months; *p* = 0.007)

Time to metastasis in M0 patients is shown in Fig. 1a. Twenty-eight patients (82.4% of metastatic patients) developed metastasis within 4 years and 31 patients (91.2%) within 8 years. Only three patients (8.8%) developed metastasis after 8 years of follow-up. In patients with lung metastases, five out of 9 patients (55.6%) had metastasis at the initial diagnosis of MLS, and all patients were diagnosed to have metastasis within 2 years (Fig. 1b), which was significantly shorter than in patients without lung metastases (15.3 months; *p* = 0.007).

The metastasis treatment characteristics and outcomes are shown in Table 2. Twenty-four patients were undergoing local radical treatment, 19 had undergone resection, and five had undergone radical radiotherapy with ≥60 Gy including carbon ion and photon beam therapy. Two patients had undergone semi-radical radiotherapy with ≥40 Gy. As a result of local radical or semi-radical treatment, 26 patients had become NED. Among 26 NED patients, 9 patients received chemotherapy for the metastasis. Disease status at final follow-up was DOD in 24 (50.0%), AWD in 14 (29.2%), and NED in 10 (20.8%) patients. There was no NED patient at the final follow-up among patients with multiple metastases. Five patients had maintained NED for over 3 years at the final follow-up.

**Table 2:** Treatment characteristics and outcomes (*N* = 48)

Variable		n		%
*No evidence of disease (NED*^a^*) following local treatment.*				
yes		**26**		54.2
	**radical treatment**		**24**	**50.0**
	resection only		12	25.0
	resection with adjuvant radiation therapy		7	14.6
	conventional radiation therapy with ≥60 Gy		2	4.2
	carbon ion therapy		2	4.2
	photon beam therapy		1	2.1
	**semi-radical treatment**		**2**	**4.2**
	conventional radiation therapy with ≥40 Gy		2	4.2
no		**22**		**45.8**
	**palliative treatment**		**20**	**41.7**
	**no treatment**		**2**	**4.2**
*Adjuvant chemotherapy for NED*^a^ *patients (n = 26)*				
yes		9		34.6
no		17		65.4
*Disease status at the final follow up*				
DOD (Dead of disease)		24		50.0
AWD (Alive with disease)		14		29.2
NED^b^ (No evidence of disease)		10		20.8

^a^NED: Status after completion of radical or semi-radical treatment for all of the remaining tumor

^b^NED: Status with no active disease detectable by imaging inspection

**Fig. 2:**
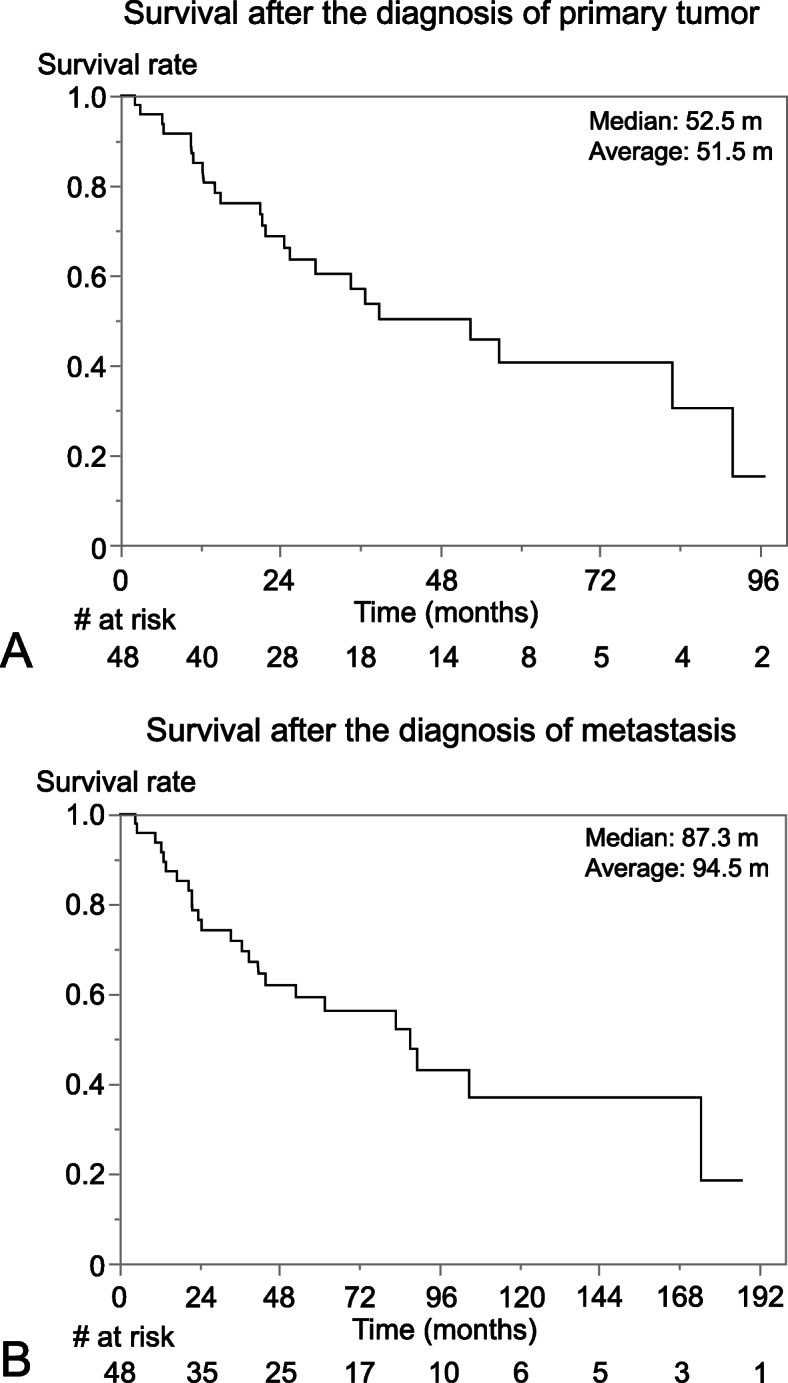
Disease-specific survival. **a** after the diagnosis of metastasis, (B) after the diagnosis of primary tumor**. a** The median disease-specific survival following the diagnosis of metastasis was 52.5 months and the 5-year survival rate was 40.6%. **b** The median disease specific survival following the diagnosis of the primary tumor for metastatic patients was 87.3 months and the 5-year survival rate was 56.2%

The median disease-specific survival following the diagnosis of metastasis was 52.5 months and the 5-year survival rate was 40.6% (Fig. 2a). The median disease specific survival following the diagnosis of the primary tumor for metastatic patients was 87.3 months and the 5-year survival rate was 56.2% (Fig. 2b).

**Fig. 3:**
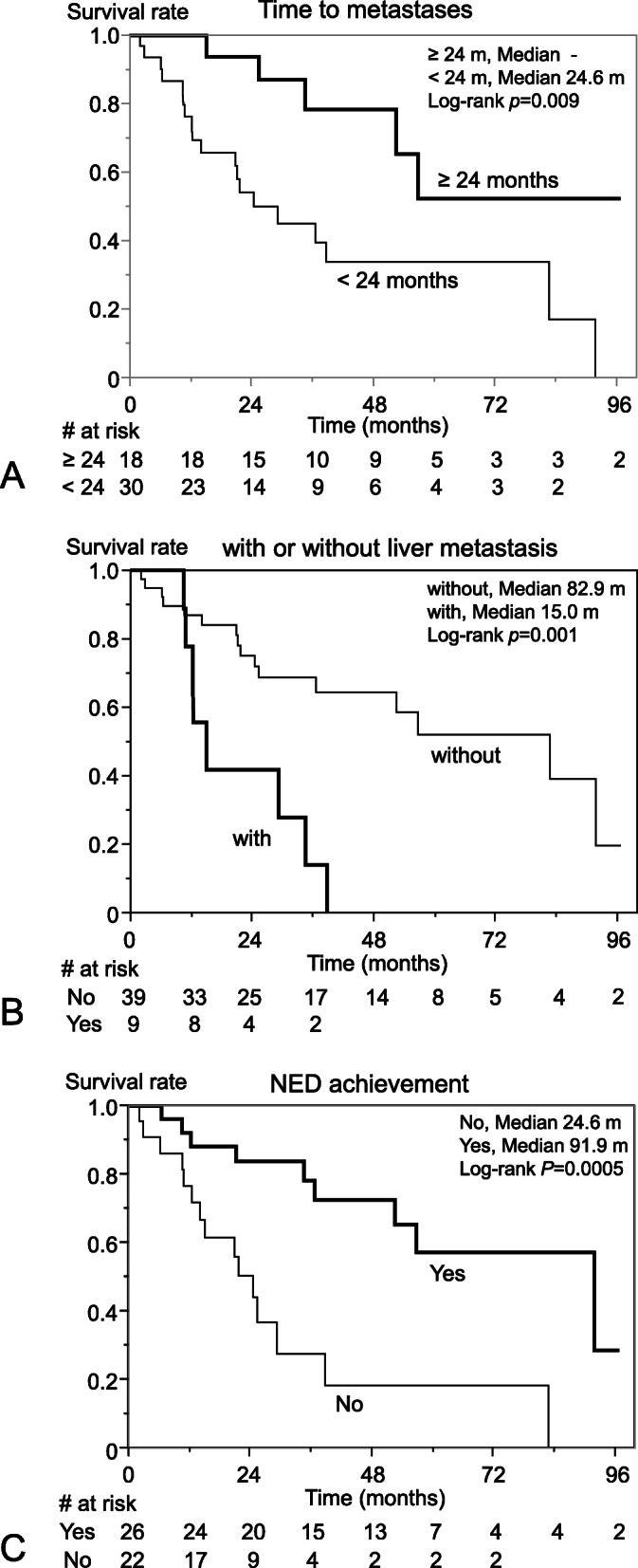
Disease-specific survival after the diagnosis of metastasis by (**a**) Time to metastasis, (**b**) with or without liver metastasis, (**c**) NED achievement. **a** Time to metastasis ≥24 month, **b** Patients without liver metastasis, and **c** NED achievement were significantly related to longer disease specific survival analyzed by Kaplan-Meier method

Next, we analyzed the prognostic factors at the initial diagnosis of metastasis (Table 3). Univariate analysis indicated that shorter time to metastasis (*p* = 0.019) and presence of liver metastasis (*p* = 0.005) were significantly related to worse prognosis. NED following local treatment was significantly related to better prognosis (*p* = 0.0007). Moreover, the survival curve examined using the Kaplan-Meier method showed a significant difference in survival for these three factors (Fig. 3a– c). Based on multivariable analysis, disease specific survival was significantly shorter in patients with liver metastases (hazard ratio [HR], 2.71; 95% confidence interval [CI], 1.00–7.09; *p* = 0.049), and longer in patients who achieved NED following local treatment (HR, 0.36; 95%CI, 0.13–0.95; *p* = 0.040). Characteristics of primary tumor and local recurrence were not related to survival based on Cox-proportional hazards regression analysis (Supplementary Table. 2).

**Table 3:** Prognostic factors for disease-specific survival after diagnosis of metastasis

(Cox-proportional hazards regression analysis)						
		Univariate			Multivariable	
Variable	HR	95%CI	*p-value*	HR	95%CI	*p-value*
Male	2.18	0.80–5.92	0.13	–	–	–
Age (years)^a^	1.02	0.99–1.06	0.24	–	–	–
Time to metastasis^‡^ (months)^a^	0.98	0.96–1.00	0.019*	0.99	0.97–1.00	0.17
Number of metastases^a^	1.18	0.97–1.42	0.10	–	–	–
Size of largest metastasis (cm)^a^	1.08	0.96–1.20	0.18	–	–	–
Location of metastasis						
lung (n = 9)	2.28	0.87–5.39	0.09	–	–	–
bone (*n* = 15)	1.01	0.41–2.32	0.97	–	–	–
abdomen or retroperitoneum (*n* = 11)	1.65	0.59–4.02	0.32	–	–	–
liver (*n* = 9)	4.14	1.60–10.2	0.005**	2.71	1.00–7.09	0.049*
NED^b^ following local treatment (n = 26)	4.45	1.86–11.5	0.0007**	0.36	0.13–0.95	0.040*
Chemotherapy for NED^b^ patients (*n* = 9)	1.95	0.52–7.30	0.32	–	–	–

^a^calculated by unit hazard ratio, ^‡^Interval from primary diagnosis to the first metastasis. It was defined as 0 month in M1 patients

^b^NED: Status after completion of radical or semi-radical treatment for all of the remaining tumor

* *p* <  0.05, **p < 0.01

Abbreviations: *HR* hazard ratio; *CI* confidence interval

Subsequently, we analyzed the factors related to NED achievement (Table 4). Univariate analysis indicated that the number of metastases (*p* <  0.0001), size of the largest metastasis (*p* = 0.04), and abdominal/retroperitoneal metastasis (*p* = 0.001) were the significant factors inhibiting NED achievement. Multivariable analysis demonstrated that number (odds ratio [OR], 0.44; 95%CI, 0.25–0.78; *p* = 0.0007) and abdominal/retroperitoneal metastases (OR, 0.99 [0.008–0.95]; *p* = 0.002) were significant factors inhibiting NED achievement.

**Table 4:** Factors for achievement of NED^a^ following local treatment (Logistic regression analysis)

	Univariate		Multivariable	
Variable	OR (95%CI)	*P*-value	OR (95%CI)	*P*-value
Age (years)^‡^	1.02	0.33	–	–
Time to metastasis^b^ (months)^‡^	1.02	0.08	–	–
Number of metastases^‡^	0.45	< 0.0001**	0.44 (0.25–0.78)	0.0007**
Size of metastasis (cm)^‡^	0.85	0.04*	0.86 (0.71–1.04)	0.11
Location of metastasis				
lung (n = 9)	0.35 (0.08–1.56)	0.27	–	–
bone (n = 15)	0.43 (0.12–1.51)	0.22	–	–
abdomen or retroperitoneum (n = 11)	0.048 (0.005–0.42)	0.001**	0.09 (0.008–0.95)	0.002**
liver (*n* = 9)	0.62 (0.14–2.66)	0.71	–	–

^a^NED: Status after completion of radical or semi-radical treatment for all of the remaining tumor

^b^Interval from primary diagnosis to the first metastasis. It was defined as 0 month in M1 patients

^‡^calculated by unit odds ratio, *p < 0.05, **p < 0.01

## Discussion

The Prognostic factors of MLS at the initial diagnosis have been described in previous studies. However, to date, the prognostic factors for metastatic MLS patients have not been elucidated in detail. In this retrospective study, we analyzed 48 metastatic MLS patients, examined the clinical course, and investigated the prognostic factors at the initial diagnosis of metastasis. Multivariable analysis indicated that liver metastases and NED achievement following radical or semi-radical local treatment for metastasis, including resection and radiation, were significant factors for longer survival. Number of metastases and abdominal/retroperitoneal metastases were the significant inhibitory factors for achieving NED. To the best of our knowledge, this is the first study to statistically demonstrate the importance of achieving NED for longer survival in metastatic MLS patients.

We first examined the epidemiology of the metastases and found that extrapulmonary metastasis was detected in 93.8% of metastatic MLS patients, which is as high as proportion reported to date. Previous reports have shown MLS metastasizes to extrapulmonary sites, including the abdominal wall and cavity, retroperitoneum, subcutaneous soft tissue, and bone, at a rate as high as 86.5% in metastatic patients [1, 3, 8, 10, 15, 25]. There has been no consensus about the mechanism why MLS tends to metastasize extrapulmonary. Asano et al. [3] have suggested that large tumor size and low histological grade are significantly associated with extrapulmonary metastasis; however, Haniball et al. [10] have reported that there is no clear correlation between the site of first metastases, tumor size, or the round cell component of the primary tumors. Our data indicates that lung metastases appear significantly earlier than extrapulmonary metastases (Fig. 1b), indicating that the mechanism of metastasis differs between pulmonary and extrapulmonary metastases or that MLS which metastasize to the lung are more aggressive than those that do not. Further investigation is needed to elucidate the mechanisms underlying the tendency of MLS to metastasize extrapulmonary, as well as the differences between pulmonary and extrapulmonary metastases, to ensure proper management of MLS metastases.

In the present study, the liver was the only significant metastatic site for poor prognosis in univariate and multivariable analyses and the survival tended to be shorter in patients with pulmonary metastases in univariate analysis (*p* = 0.09). Previous reports have shown that the disease-free interval and the overall survival rate were significantly better in patients with extrapulmonary metastases compared to those with pulmonary metastases [3, 6, 17]. Liver metastasis has not been reported to be important for survival, as these previous studies did not analyze the prognosis by each metastatic organ. Taken together, our current results and these previous reports suggest that liver and lung metastases may be prognostic factors of shorter survival. As vital organ such as brain, lung, or liver metastasis is associated with poor prognosis in common cancers [13], it is plausible that lung and liver metastasis may also be related to poor prognosis in MLS patients. In addition, the poor prognosis of patients with lung metastases might be due to more aggressive biology or the speed, as discussed in the previous paragraph.

In this study, NED achievement was one of the significant factors related to disease specific survival in multivariable analysis. A number of reports support our findings. Spillane et al. [24] analyzed the natural history of soft tissue metastasis from MLS and concluded that soft tissue metastases should be managed aggressively, most often involving further surgery, because patients only with soft tissue metastasis have relatively long prognosis. There are two case reports of long-term survival following complete resection of metastases [22, 26]. Although extrapulmonary metastasis has been thought to be equivalent to systemic metastasis in all types of sarcomas, long-term survival has been achieved when a complete resection was possible for both the pulmonary and extrapulmonary metastases [4]. Our results and these reports indicate that complete resection of metastases from MLS should be considered, if possible.

We had hypothesized that complete resection of metastases might lead to the best prognosis; however, NED achievement by resection was not significant in the present study (data not shown). As MLS is known to be particularly sensitive to radiotherapy compared with other histological subtypes of soft tissue sarcoma [7, 18], we included patients who were administered radical and semi-radical radiotherapy for the metastasis, which significantly improved the prognosis of NED patients. In addition to patients who underwent radical radiation therapy, including carbon ion therapy, photon beam therapy, and conventional radiation therapy with ≥60 Gy, there were two patients who achieved NED by semi-radical radiation therapy with ≥40 Gy. In both cases there was no local recurrence surrounding the irradiated metastasis at the final follow up, indicating that semi-radical radiation therapy might be useful for extending survival when radical therapy cannot be adapted. Based on these data, the first choice treatment strategy should be metastasis resection; however, if this is not possible, radiotherapy might be the second choice, as this could lead to a better prognosis. Further investigation is needed with a larger number of patients to determine the effect of radiotherapy on survival.

The number of metastases and metastases to abdomen or retroperitoneum were the significant inhibitory factor for achieving NED. As the number was significant factor, it might be better to detect metastases while the number is low by periodic surveillance. Total body MRI has been proposed for early detection of bone and soft tissue metastases, including MLS metastasis [19, 21]. However, total body MRI is not common in Japan or around the world. Importantly, both PET scans and bone scans are reported as not highly sensitive to MLS metastases [20, 21]. In the present study, the metastases of 35 (73.1%) patients were detected by CT. As CT is one of the most common imaging modalities, liver metastasis was one of the worse prognostic factors by multivariable analysis, and abdominal/retroperitoneal metastases were factors inhibiting the achievement of NED, contrast-enhanced CT might be useful for metastasis surveillance in MLS patients. In the present study, the number of patients who developed metastasis linearly increased to 80% within the first 4 years and approximately 90% of the metastatic patients were diagnosed within 8 years (Fig. 1a). Thus, we recommend whole body surveillance 2–3 times per year during the first 4 years and once a year till 8 years. As the presence of a round cell component on histopathology was reported to be predictive of a much higher metastatic rate compared with the absence of a round cell component [14, 23, 24], surveillance should be recommended especially for patients with a round cell component > 5%. It should also be noted that a number of patients will develop metastases beyond the 8 years after the initial diagnosis. As it is not pragmatic to survey all patients with whole body CT once a year after 8 years of follow-up, we should carefully listen to patients over 15 years, check whether there are symptoms or signs of recurrence, and perform contrast-enhanced CT if any appear.

This study had some limitations. First and most importantly, this was a retrospective study. Although we showed that achievement of NED was the important prognostic factor in multivariable analysis, it is possible that the tumors of NED patients might have been less aggressive originally. In addition, modality and frequency of imaging examination for systemic screening before and after the diagnosis of metastasis were not completely unified. Different imaging modalities could lead to variable results. Second, we did not investigate the proportion of the round cell component. Although previous study which analyzed 160 patients reported that survival following the diagnosis of metastasis was not affected by the round cell component [10], it may be still important and has to be confirmed in the future. Third, we did not examine the effect of palliative chemotherapy. In this study, as we investigated the prognostic factors at the initial diagnosis of metastasis, palliative chemotherapy was performed only for patients without NED. Because all of the patients with metastasis including NED patients at the initial diagnosis would receive palliative chemotherapy when metastases get locally uncontrollable, we thought we would not be able to see the effect. However, trabectedin, the effect of which is apparent for MLS, was not used in daily practice because it had not been covered by insurance in Japan during the study registration period.

## Conclusions

Over 90% of MLS metastatic patients had extrapulmonary metastases. Although only 18.8% had lung metastasis, it appeared significantly earlier than extrapulmonary metastases. As multivariable analysis indicated that NED achievement following resection or radiation therapy were significant factors for longer survival, resection or radiation therapy should be considered for all of the metastases. As number of metastases was a significant factor for achieving NED, early detection of metastases might be important.

## Supplementary information

**Additional file 1 Supplementary Table 1.** Characteristics and treatment of primary tumor (*N* = 48).

**Additional file 2 Supplementary Table 2.** Prognostic factors regarding primary tumor for disease-specific survival after diagnosis of metastasis. (Cox-proportional hazards regression analysis).

## Data Availability

The datasets used and analyzed in the current study are available from the corresponding author upon reasonable request.

## Abbreviations

MLS: Myxoid liposarcoma
NED: No evidence of disease
AWD: Alive with disease
DOD: Dead of disease
CT: Computed tomography
MRI: Magnetic resonance imaging
PET: Positron emission tomography

## References

[1] Antonescu CR, Ladanyi M, Fletcher CDM, Unni KK, Mertens F (2002). Myxoid liposarcoma. World Health Organization classification of Tumours: pathology and genetics of Tumours of soft tissue and bone.

[2] Antonescu CR, Tschernyavsky SJ, Decuseara R (2001). Prognostic impact of P53 status, TLS-CHOP fusion transcript structure, and histological grade in myxoid liposarcoma: a molecular and clinicopathologic study of 82 cases. Clin Cancer Res.

[3] Asano N, Susa M, Hosaka S (2012). Metastatic patterns of myxoid/round cell liposarcoma: a review of a 25-year experience. Sarcoma.

[4] Blackmon SH, Shah N, Roth JA (2009). Resection of pulmonary and extrapulmonary sarcomatous metastases is associated with long-term survival. Ann Thorac Surg.

[5] Chang HR, Hajdu SI, Collin C, Brennan MF (1989). The prognostic value of histologic subtypes in primary extremity liposarcoma. Cancer..

[6] Cheng EY, Springfield DS, Mankin HJ (1995). Frequent incidence of extrapulmonary sites of initial metastasis in patients with liposarcoma. Cancer..

[7] Chung PW, Deheshi BM, Ferguson PC (2009). Radiosensitivity translates into excellent local control in extremity myxoid liposarcoma: a comparison with other soft tissue sarcomas. Cancer..

[8] Enzinger FM, Weiss SM, Enzinger FM, Weiss SM (2001). Liposarcoma. *Soft Tissue Tumors*: St.

[9] Fiore M, Grosso F, Vullo SL (2007). Myxoid/round cell and pleomorphic liposarcomas: prognostic factors and survival in a series of patients treated at a single institution. Cancer.

[10] Haniball J, Sumathi VP, Kindblom LG (2011). Prognostic factors and metastatic patterns in primary myxoid/round-cell liposarcoma. Sarcoma.

[11] Hashimoto H, Enjoji M (1982). Liposarcoma. A clinicopathologic subtyping of 52 cases. Acta Pathol Jpn.

[12] Heuvel SET, Hoekstra HJ, Ginkel RJV, Bastiaannet E, Suurmeijer AJH (2007). Clinicopathologic prognostic factors in myxoid liposarcoma: a retrospective study of 49 patients with long-term follow-up. Ann Surg Oncol.

[13] Katagiri H, Okada R, Takagi T (2014). New prognostic factors and scoring system for patients with skeletal metastasis. Cancer Med.

[14] Kilpatrick SE, Doyon J, Choong PFM, Sim FH, Nascimento AG (1996). The clinicopathologic spectrum of myxoid and round cell liposarcoma: a study of 95 cases. Cancer..

[15] Meis-Kindblom J, Sjogren H, Kindblom LG (2001). Cytogenetic and molecular genetic analyses of liposarcoma and its soft tissue simulators: recognition of new variants and differential diagnosis. Virchows Arch.

[16] Moreau LC, Turcotte R, Ferguson P (2012). Myxoid/round cell liposarcoma (MRCLS) revisited: an analysis of 418 primarily managed cases.; Canadian Orthopaedic oncology society (CANOOS). Ann Surg Oncol.

[17] Pearlstone DB, Pisters PW, Bold RJ (1999). Patterns of recurrence in extremity liposarcoma: implications for staging and follow-up. Cancer.

[18] Pitson G, Robinson P, Wilke D (2004). Radiation response: an additional unique signature of myxoid liposarcoma. Int J Radiat Oncol Biol Phys.

[19] Schwab JH, Boland PJ, Antonescu C (2007). Spinal metastases from myxoid liposarcoma warrant screening with magnetic resonance imaging. Cancer..

[20] Schwab JH, Healey JH (2007). FDG-PET lacks sufficient sensitivity to detect myxoid liposarcoma spinal metastases detected by MRI. Sarcoma.

[21] Sheah K, Ouellette HA, Torriani M (2008). Metastatic myxoid liposarcomas: imaging and histopathologic findings. Skelet Radiol.

[22] Shi X, Matsumoto S, Manbe J (2006). Long-term survival of soft tissue sarcoma patients with extrapulmonary metastasis. J Orthop Sci.

[23] Smith TA, Easley KA, Goldblum JR (1996). Myxoid/round cell liposarcoma of the extremities: a clinicopathologic study of 29 cases with particular attention to extent of round cell liposarcoma. Am Journal of Surg Pathol.

[24] Spillane AJ, Fisher C, Thomas JM (1999). Myxoid liposarcoma--the frequency and the natural history of nonpulmonary soft tissue metastases. Ann Surg Oncol.

[25] Tos APD (2000). Liposarcoma: new entities and evolving concepts. Ann Diag Pathol.

[26] Yokouchi M, Nagano S, Kijima Y (2014). Solitary breast metastasis from myxoid liposarcoma. BMC Cancer.

