# Supplementary material for: Prognostic factors of metastatic myxoid liposarcoma
Source: BMC Cancer. 2020 Sep 14;20:883. doi: 10.1186/s12885-020-07384-1 (PMC7491192; doi:10.1186/s12885-020-07384-1)
Supplement: Supplementary file 1 — Additional file 1 Supplementary Table 1. Characteristics and treatment of primary tumor (N = 48). [file 12885_2020_7384_MOESM1_ESM.docx]

Supplementary Table 1. Characteristics and treatment of primary tumor (N = 48)

| Variable | n | % |
| --- | --- | --- |
| *Age at diagnosis of primary tumor* [median: 41 years (26 – 71)] | |  |
| < 45 | 21 | 43.8 |
| ≥ 45 years | 27 | 56.2 |
| *Location* |  |  |
| Thigh | 33 | 68.8 |
| Trunk | 7 | 14.6 |
| Others | 8 | 16.7 |
| *Size* [median: 13 cm (5 – 27)] |  |  |
| < 10 cm | 32 | 66.6 |
| ≥ 10 | 11 | 22.9 |
| unknown | 5 | 10.4 |
| *Chemotherapy for primary tumor (in M0 patients; n = 34)* | |  |
| Yes | 6 | 17.6 |
| No | 28 | 82.4 |
| *Local treatment for primary tumor* |  |  |
| M0 patients (n = 34) |  |  |
| resection | 34 | 100.0 |
| M1 patients (n = 14) |  |  |
| resection | 7 | 50.0 |
| radiation | 3 | 21.5 |
| no treatment | 4 | 28.5 |
| *Local recurrence at the initial diagnosis of metastasis (in M0 patients; n = 34)* | | |
| Yes | 5 | 14.7 |
| No | 22 | 64.7 |
