# Supplementary material for: Prognostic factors of metastatic myxoid liposarcoma
Source: BMC Cancer. 2020 Sep 14;20:883. doi: 10.1186/s12885-020-07384-1 (PMC7491192; doi:10.1186/s12885-020-07384-1)
Supplement: Supplementary file 2 — Additional file 2 Supplementary Table 2. Prognostic factors regarding primary tumor for disease-specific survival after diagnosis of metastasis. (Cox-proportional hazards regression analysis). [file 12885_2020_7384_MOESM2_ESM.docx]

Supplementary Table 2. Prognostic factors regarding primary tumor for disease-specific survival after diagnosis of metastasis. (Cox-proportional hazards regression analysis)

|  |  | Univariate |  |
| --- | --- | --- | --- |
| Variable | HR | 95%CI | *p-value* |
| Age at diagnosis of primary tumor (years )^†^ | 1.03 | 0.99 – 1.06 | 0.10 |
| Size of primary tumor (cm)^†^ | 0.98 | 0.91 – 1.06 | 0.71 |
| Location of primary tumor |  |  |  |
| thigh (n=33) | 1.07 | 0.47 – 2.63 | 0.88 |
| trunk (n=7) | 1.06 | 0.31 – 3.65 | 0.92 |
| Local recurrence at the initial diagnosis of metastasis in M0 patients (n=5) | 1.11 | 0.23 – 5.28 | 0.89 |
| ^†^ calculated by unit hazard ratio, * *p*<0.05 |  |  |  |
| Abbreviations: HR, hazard ratio; CI, confidence interval |  |  |  |
